# N2A, MCF7, and HepG2 Cells Support Intracellular Replication of *Coxiella burnetii*

**DOI:** 10.17912/micropub.biology.001716

**Published:** 2025-08-13

**Authors:** Stephen M Kotfila, Sarah J Leduc, Genna M Mullen, Shawna C O Reed

**Affiliations:** 1 Department of Biomedical Sciences, Quinnipiac University; 2 Department of Biomedical Sciences, Quinnipiac University, Hamden, Connecticut, United States

## Abstract

*Coxiella burnetii* is a gram-negative, obligate, intracellular bacterial pathogen that causes zoonotic Q fever in humans. In a mammalian host, *C. burnetii* may infect macrophage, heart, brain, liver, and placental cells. *C. burnetii* is routinely cultured in HeLa (human cervical) and Vero (African green monkey kidney) cells for research, but these cell types poorly reflect the natural replicative niche. Here we report the first evidence of *C. burnetii* , Nine Mile Phase II, replication in N2A (mouse neuronal), HepG2 (human hepatocyte), and MCF7 (human mammary epithelial) cells. These findings will enable further comparative study of *C. burnetii* cytopathology and infection dynamics in various cell types.

**Figure 1. C. burnetii Establishes Infection in HeLa, N2A, MCF7, and HepG2 Cells:**
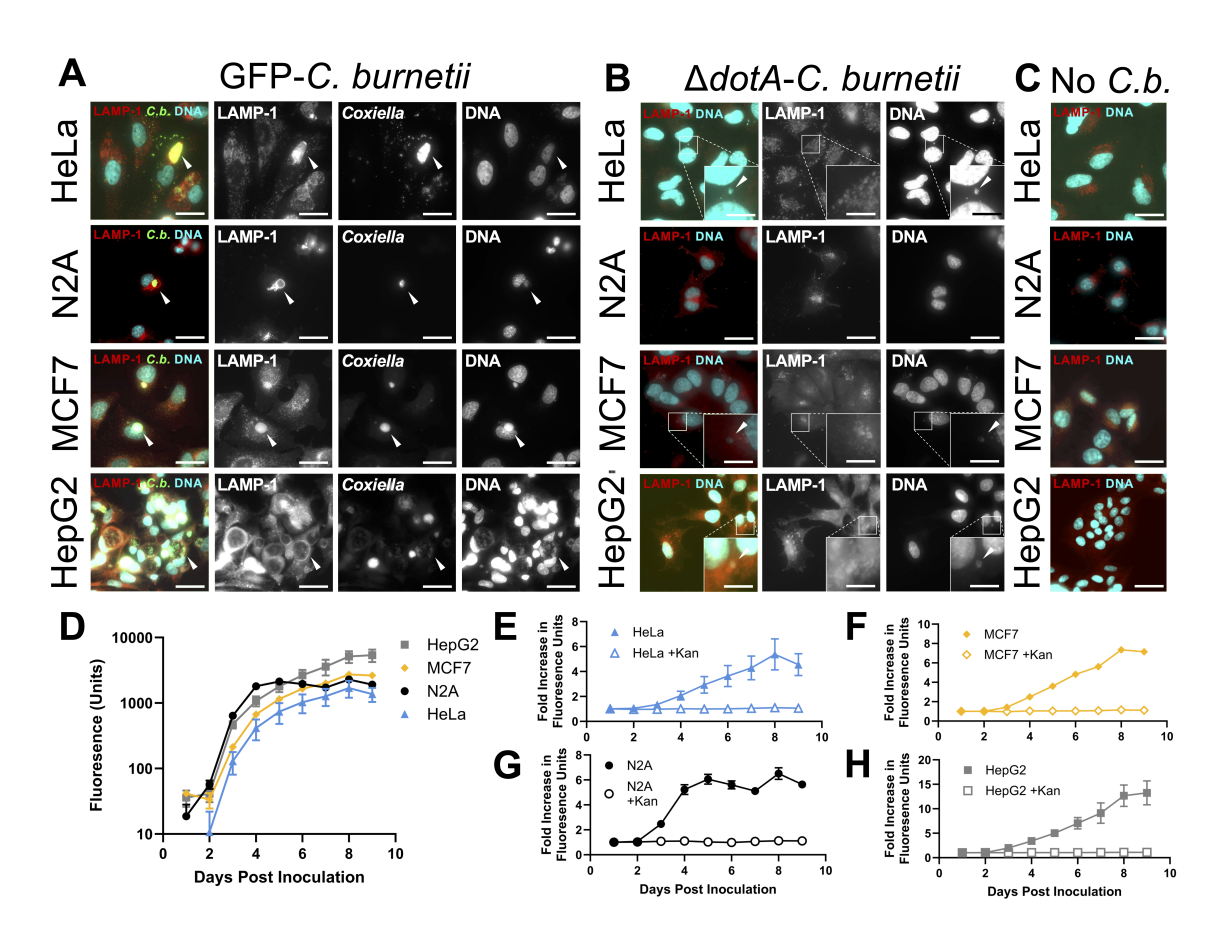
(A) *GFP-C. burnetii* infected, (B) *dotA::tn* - *C. burnetii* infected, and (C) uninfected HeLa, N2A, MCF7, and HepG2 cells at 60X magnification. Green, GFP fluorescence of bacteria; Red, anti-mouse (N2A) or anti-human LAMP-1 antibody; Cyan, Hoechst 33342 DNA staining. In GFP- *C. burnetii* infected cells (A), white arrowheads indicate *Coxiella* vacuoles (A) or individual bacteria in *dotA::tn* infected cells (B). Scale bar: 30µm or 10µm in zoom. (D) Background-adjusted fluorescence values and fold change in fluorescence values, relative to day one measurements, of GFP- *C. burnetii* infected (E) HeLa, (F) MCF7, (G) N2A, and (H) HepG2 cells with or without 75µg/mL of kanamycin. (D-H) Data points: mean ± SEM two biological replicates; three technical replicates each.

## Description

*Coxiella burnetii* is a pathogenic rickettsial coccobacillus in the class Gammaproteobacteria (Celina & Cerný, 2022; Shaw & Voth, 2019). *C. burnetii* is obligately intracellular in its natural infection cycle, and may be transmitted to humans from infected domestic animals, causing zoonotic Query fever disease (Q fever) in humans (Celina & Cerný, 2022; Shaw & Voth, 2019; Voth & Heinzen, 2007). Transmission to humans occurs primarily through the inhalation of materials contaminated by parturition products of infected goats, sheep or camels (Celina & Cerný, 2022; Christian, 2013; Duron et al., 2015). Q fever may be an acute, self-limiting febrile illness with occasional pneumonia or hepatitis or may chronically infect humans, often resulting in endocarditis (Maurin & Raoult, 1999). *C. burnetii* infection has also been associated with rare encephalitis (Brooks et al., 1986; Lim et al., 2014). Granulomatous hepatitis, endocarditis, and other complications of acute or chronic Q fever are common and severe in immunocompromised and pregnant patients (Riechman et al., 1988). To conduct research in the absence of restrictive select agent and biosafety guidelines, an avirulent strain, Nine Mile phase II, with a truncated LPS molecule, is routinely used for *in vitro* studies (Voth & Heinzen, 2007).

Growth of *C. burnetii* requires glucose or gluconeogenic precursors and a mixture of amino acids at a low pH (Shaw & Voth, 2019; Vallejo Esquerra et al., 2017). These requirements are satisfied in axenic (cell-free) culture using ACCM-2 media (Omsland et al., 2009). In an infected cell, these conditions are present in the acidified late endosome formed after a cell engulfs a bacterium. Subsequently, the late endosome fuses with lysosomes, where amino acid transporters are necessary to satisfy the amino acid requirements and promote *Coxiella* growth (P. Newton et al., 2020; Steiner et al., 2021; van Schaik et al., 2013). The mature *Coxiella* -containing vacuole (CCV) is an expansive compartment that supports *Coxiella* replication, fuses with other CCVs in the cell, and is decorated with lysosomal and autophagosomal markers such as LAMP-1and LC3-II (Kohler & Roy, 2015)

*C. burnetii* expresses a dot/icm type IV secretion system which translocates bacterial effector proteins across both bacterial membranes and across the vacuolar membrane to hijack host cell machinery and promote replication (Carey et al., 2011; Voth et al., 2009). Insertional *dot/icm* mutant strains ( *dotA::tn)* of *C. burnetii* cannot translocate effectors which prevents productive infection (H. J. Newton et al., 2014). Host factors are also important for survival within infected cells, especially the transcription factors TFEB and TFE3, retromer complex, Rab7, and amino acid transporters at the lysosome, and are required to support the formation of the spacious CCV (Killips et al., 2024; McDonough et al., 2013; P. Newton et al., 2020; Padmanabhan et al., 2020). *Legionella pneumophila* , a close relative of *C. burnetii* , also exploits a host cell solute carrier (SLC) 1A5 to translocate amino acids into the intravacuolar space (Miller et al., 2019). These conditions are satisfied by the intracellular environments of HeLa (human cervical epithelial), Vero (African Green Monkey kidney epithelial), and THP-1 (human monocyte-like) cell lines, which are routinely used to propagate and study *C. burnetii* (Lockhart et al., 2013; Wan et al., 2023). *C. burnetii* has also been propagated in HEK-293T (human embryonic kidney), DH82 (canine macrophage-like), L929 (murine fibroblast), and XTC-2 (African clawed frog fibroblast) cells (Duncan-Lowey et al., 2023; Lockhart et al., 2013).

There are no previously published reports of *C. burnetii* replication in hepatocyte, neuron, or mammary epithelial cell lines *in vitro* , despite these tissues’ clinical significance. As part of a course-based undergraduate research experience (CURE), we sought to investigate the growth of *C. burnetii* NMII in well-characterized N2A (mouse neuronal), MCF7 (human mammary epithelial), and HepG2 (human hepatocyte) cells. Based on the expression of TFEB and TFE3 and solute carriers, such as SLC-1A5, in these tissue types we anticipated intracellular replication of *C. burnetii* NMII in all cell lines, but were interested in how growth would compare to HeLa cells more commonly used for *Coxiella* research (El-Houjeiri et al., 2021; Gray et al., 2004; Li et al., 2018; Thompson et al., 2014; Uhlen et al., 2010; Uhlén et al., 2015; Visel, 2004) We infected each cell line with either a GFP-expressing wild type clone of *C. burnetii* NMII or the NMII *dotA::tn* mutant at an approximate multiplicity of infection of 250.

We observed intracellular replication of *C. burnetii* NMII in all cell lines (Figure 1). Productive infection was evidenced by clear intravacuolar compartmentalization of GFP- *C. burnetii* ( Figure 1A ). These vacuoles were decorated with LAMP-1 protein and contained microcolonies of replicating bacteria ( Figure 1A ). In the *dotA::tn* - *C. burnetii* infected cells, LAMP-1 was distributed in small vesicles throughout the cell and no large CCVs were formed ( Figure 1B ). Uninfected cells displayed an even distribution of LAMP-1 decorated vesicles ( Figure 1C ).

Intracellular replication of GFP- *C. burnetii* varies among the cell lines, as indicated by different raw fluorescence values and fold change values across a nine day infection ( Figure 1D- E). Our growth curve data suggests that HepG2 cells support the most replication of *C. burnetii* over a nine day period, while N2A cells boast the fastest growth, stalling by around day 5 post-infection ( Figure 1D, G, H). In each growth curve, the addition of a selective concentration of kanamycin (75µg/mL) inhibited increase in green fluorescence, confirming that increase in fluorescence was indeed due to bacterial growth and was not autofluorescence as a result of cell death ( Figure 1E- H).

Based on microscopy of stained cells, HepG2 cells supported the largest CCVs relative to the whole cell size ( Figure 1A ). The size and morphology of CCVs may be due to differential expression of host cell proteins such as the lysosomal transcription factors TFEB and TFE3, which both support and restrict spacious CCV formation depending on the cellular context (Killips et al. 2024, Padmanabhan et al., 2020). It would be interesting to investigate global transcriptional response to infection in each cell line leading to identification of these and other pathways differentially activated in response to *C. burnetii* infection in various cell types. Another possible explanation for the growth and CCV formation in HepG2 cells is cholesterol and lipid metabolism. HepG2 cells are known to possess a plethora of lipid droplets and lipid droplet metabolism has been demonstrated to promote intracellular growth and survival of *C. burnetii* (Biancaniello & Mulye, 2024; Gao & Goodman, 2015; Mulye et al., 2018) *.* Finally, our observations that mouse neuronal cells support bacterial replication (Figure 1) suggests *C. burnetii* may be capable of intraneuronal growth *in vivo* . It would be interesting to investigate intracellular replication of *C. burnetii* in a human neuronal cell line.

In future studies, absolute quantification of replication using genome equivalent (qPCR) assays or colony forming unit assays could be compared to investigate whether bacterial fluorescence is an indicator of viable growth in various cell lines. In addition, these methods could be used in other primary, human derived cells to better reflect the full innate immune barriers to bacterial infection of terminally differentiated cells. Overall, our work expands the repertoire of cells available to study *Coxiella* infection and provide methods useful for future investigations. Using a human neuronal cell line may reveal different growth characteristics than the mouse cell line used in this work.

## Methods

**Mammalian and Bacterial Cell Culture**

Cultures of mammalian cells were maintained in 10% Fetal Plus FBS (Atlas Biological) and DMEM with GlutaMAX and penicillin/streptomycin (Gibco/Thermo Fisher). *Coxiella burnetii* Nine Mile, Phase II bacteria were grown in ACCM-2 medium (Sunrise Science) with GlutaMAX and 0.5mM supplemental L-tryptophan.

**Growth Curves**

Two days prior to infection, culture medium was exchanged for DMEM containing 5% Fetal Select (Atlas Biological) and without antibiotics. On the day of infection, cells were trypsinized and plated at 1x10 ^4^ cells/well (HeLa, HepG2, and N2A) or 1.25x10 ^4^ cells/well (MCF7) in a 96 well plate, to achieve a confluency of about 80%, and mixed in triplicate wells with 2.5x10 ^6^ GE (HeLa, HepG2, and N2A) or 3.13x10 ^6^ GE (MCF7) of *C. burnetii* (att::GFP) to achieve a MOI of 250; in parallel, three wells also contained kanamycin (75µg/mL). Cells were centrifuged at 172xg at 22ºC for five minutes, prior to incubation at 5% CO _2_ and 37ºC. 24 hours post-infection, cells were rinsed twice with 1X Hank’s Balanced Salt Solution (HBSS) and medium was exchanged for FluoroBrite DMEM (Thermo Fisher Scientific) with 2% FBS (Atlas Biological; FS-0500-AD) and kanamycin for antibiotic-treated wells. On days 1-9, fluorescence was measured using a Tecan Infinite M200 plate reader, excitation wavelength of 500nm, emission 535nm, 200μm border, 4x4 circle (filled) read pattern and a gain of 150.

**Immunofluorescence and Microscopy**

Cells were cultured as above and plated at 1 × 10 ^5^ cells/well (HeLa, HepG2, and N2A) and 1.25x10 ^5^ cells/well (MCF7) in a 24 well plate with glass 12mm coverslips. 2.5x10 ^7^ GE/well (HeLa, HepG2, and N2A) and 3.13x10 ^7^ GE/well (MCF7) of GFP and dota::tn *C. burnetii* (GFP) were mixed with the cell suspension. Three days post-infection, cells were washed with 1x HBSS and the media was exchanged. 5 days after infection, cells were fixed with 4% Paraformaldehyde in HBSS for 20 minutes and stored until staining. Coverslips were permeabilized in 0.5% Saponin in 1X PBS for five minutes, blocked in 2% BSA, 0.1% Triton, and 1X PBS for one hour, stained with mouse anti-human LAMP1 or rat anti-mouse LAMP1 antibodies (diluted 1:500 in block) for one hour, rinsed and incubated with Anti-Mouse Dylight 594 secondary or Anti-Rat Alexa 555 secondary (diluted 1:500) and Hoechst 33342 (diluted 1:2,000 in block) solution for 45 minutes. The coverslips were then rinsed and mounted using Prolong Glass (Invitrogen Thermo Fisher) on slides for visualization. All cell types were imaged on an ECHO Revolve microscope at 600X total magnification, oil immersion lens.

**Data Analysis**

Raw fluorescence values (see Growth Curves, above) were processed in Microsoft Excel and background adjusted by subtracting the fluorescence value of an uninfected well from GFP- *C. burnetii* infected wells for each cell type. Fold change values ( Figure 1E- H) were calculated by dividing day *x* fluorescence values by the respective day 1 fluorescence.

## Reagents

**Table:** 

**Product**	**Supplier**	**Reference**
96-well microclear plate	Greiner Bio-One	675086
DMEM, high glucose, pyruvate	Thermo Fisher	11995081
FluoroBrite DMEM	Thermo Fisher	A1896702
GlutaMax	Thermo Fisher	35050-061
Penicillin/Streptomycin	Thermo Fisher	15140148
Fetal Plus Serum	Atlas Biological	FP-0500-A
Fetal Select Bovine Serum	Atlas Biological	FS-0500-AD
L-Tryptophan	Sigma	T-0254
ACCM-2	Sunrise Science	4700-300
Saponin	Sigma Aldrich	SAE0073
Goat Anti-mouse Dylight 594	Thermo Fisher	35511
Goat Anti-rat Alexa 555	Thermo Fisher	A21434
Mouse Anti-human LAMP1	Developmental Studies Hybridoma Bank (DSHB)	H4A3
Rat Anti-mouse LAMP1	Developmental Studies Hybridoma Bank (DSHB)	1D4B

**Table:** 

**Strain**	**Genotype**	**Provided by**
NMII GFP SS400	*C. burnetii Nine Mile II, RSA439* (att::311-GFP)	Samuel Steiner and Craig Roy (Yale University School of Medicine)
NMII *dotA::tn*	*C. burnetii* ( *dotA* ::tn)	Samuel Steiner and Craig Roy (Yale University School of Medicine)

**Table:** 

**Cell Line**	**Description**	**ATCC Designation**
HepG2	Human hepatocellular carcinoma	HB-8065
Neuro-2A (N2A)	Mouse neuroblastoma	CCL-131
MCF7	Human mammary epithelial adenocarcinoma	HTB-22
HeLa	Human cervical carcinoma	CCL-2
